# Gene editing of authentic *Brassica rapa flavonol synthase 1* generates dihydroflavonol-accumulating Chinese cabbage

**DOI:** 10.1093/hr/uhad239

**Published:** 2023-11-14

**Authors:** Sangkyu Park, Hyo Lee, Jaeeun Song, Chan Ju Lim, Jinpyo Oh, Sang Hoon Lee, Saet Buyl Lee, Jong-Yeol Lee, Sunhyung Lim, Jin A. Kim, Beom-Gi Kim

**Affiliations:** Metabolic Engineering Division, National Institute of Agricultural Sciences, Rural Development Administration, Jeonju, 54874, South Korea; Metabolic Engineering Division, National Institute of Agricultural Sciences, Rural Development Administration, Jeonju, 54874, South Korea; Metabolic Engineering Division, National Institute of Agricultural Sciences, Rural Development Administration, Jeonju, 54874, South Korea; Institute of Biotechnology and Breeding, Asiaseed Inc., Icheon, 17414, South Korea; Institute of Biotechnology and Breeding, Asiaseed Inc., Icheon, 17414, South Korea; Food and Nutrition Division, National Institute of Agricultural Sciences, Rural Development Administration, Wanju, 55365, South Korea; Metabolic Engineering Division, National Institute of Agricultural Sciences, Rural Development Administration, Jeonju, 54874, South Korea; Metabolic Engineering Division, National Institute of Agricultural Sciences, Rural Development Administration, Jeonju, 54874, South Korea; Department of Horticultural Biotechnology, School of Biotechnology, Hankyong National University, Anseong, 17579, South Korea; Metabolic Engineering Division, National Institute of Agricultural Sciences, Rural Development Administration, Jeonju, 54874, South Korea; Metabolic Engineering Division, National Institute of Agricultural Sciences, Rural Development Administration, Jeonju, 54874, South Korea

## Abstract

Flavonols are the major class of flavonoids of green Chinese cabbage (*Brassica rapa* subsp. *pekinensis*). The *B. rapa* genome harbors seven *flavonol synthase* genes (*BrFLS*s), but they have not been functionally characterized. Here, transcriptome analysis showed four *BrFLS*s mainly expressed in Chinese cabbage. Among them, only BrFLS1 showed major FLS activity and additional flavanone 3*β*-hydroxylase (F3H) activity, while BrFLS2 and BrFLS3.1 exhibited only marginal F3H activities. We generated *BrFLS1*-knockout (*BrFLS1-*KO) Chinese cabbages using CRISPR/Cas9-mediated genome editing and obtained transgene-free homozygous plants without off-target mutation in the T_1_ generation, which were further advanced to the T_2_ generation showing normal phenotype. UPLC-ESI-QTOF-MS analysis revealed that flavonol glycosides were dramatically decreased in the T_2_ plants, while dihydroflavonol glycosides accumulated concomitantly to levels corresponding to the reduced levels of flavonols. Quantitative PCR analysis revealed that the early steps of phenylpropanoid and flavonoid biosynthetic pathway were upregulated in the *BrFLS1-*KO plants. In accordance, total phenolic contents were slightly enhanced in the *BrFLS1-*KO plants, which suggests a negative role of flavonols in phenylpropanoid and flavonoid biosynthesis in Chinese cabbage. Phenotypic surveys revealed that the * BrFLS1-KO* Chinese cabbages showed normal head formation and reproductive phenotypes, but subtle morphological changes in their heads were observed. In addition, their seedlings were susceptible to osmotic stress compared to the controls, suggesting that flavonols play a positive role for osmotic stress tolerance in *B.rapa* seedling. In this study, we showed that CRISPR/Cas9-mediated *BrFLS1*-KO successfully generated a valuable breeding resource of Chinese cabbage with distinctive metabolic traits and that CRISPR/Cas9 can be efficiently applied in functional Chinese cabbage breeding.

## Introduction

Flavonoids are important bioactive compounds naturally found in the plant kingdom. Therefore, the flavonoid biosynthesis pathway has been thoroughly investigated in various plant species. The first step in flavonoid biosynthesis involves condensation of *p*-Coumaroyl-CoA with three molecules of malonyl-CoA to produce chalcone, which is catalyzed by chalcone synthase (CHS). Chalcone is then subjected to intramolecular cyclization to form (2*S*)-flavanone by chalcone isomerase (CHI); (2*S*)-flavanone serves as a universal substrate for flavonoid biosynthesis, which is hydroxylated by flavanone 3*β*-hydroxylase (F3H) to form dihydroflavonols, which are further oxidized to flavonols by flavonol synthase (FLS). Dihydroflavonols can also be consecutively converted to form the anthocyanin precursors leucoanthocyanidins and anthocyanidins by dihydroflavonol 4-reductase (DFR) and anthocyanidin synthase (ANS), respectively. In another branch, (2*S*)-flavanone is converted to flavones by flavone synthase I or II (FNSI or FNSII).

Chinese cabbage (*Brassica rapa* var. *pekinensis*), widely cultivated in East Asia, is an economically important crop. Particularly, green heading Chinese cabbages are consumed as the main ingredient of kimchi. Green Chinese cabbage contains various bioactive molecules, e.g. carotenoids, glucosinolates, and phenolic compounds. Among them, phenolic compounds that exhibit characteristic antioxidants account for a large proportion. The major classes of phenolic compounds accumulating in green Chinese cabbage are flavonoids, mainly flavonols, and phenolic acids [1, 2]. In Brassicaceae, *Arabidopsis thaliana FLS* (*AtFLS*) and *Brassica napus FLS* (*BnaFLS*) families have been functionally characterized. *Arabidopsis thaliana* genome harbors six *AtFLSs* on chromosome 5, among which *AtFLS1* encodes the major active FLS enzyme [3], and *AtFLS3* marginally contributes to flavonol biosynthesis [4]. In *B. napus*, 13 candidate *FLS* genes were identified, among which *BnaFLS1–1* and *BnaFLS1–2* are known as mainly active isoforms, and *BnaFLS3–3* and *BnaFLS3–4* encode enzymes marginally exhibiting F3H activity, whereas the other members of this family were proposed to be pseudogenes [5]. *Brassica rapa*, which diverged from *A. thaliana*, has undergone tandem duplications, whole-genome triplication, polyploidization, and fractionation of its subgenomes. Consequently, homologs of *B. rapa FLS* (*BrFLS*) genes are distributed across four different chromosomes (A02, A06, A09, and A10) [6–8]. Six *BrFLS* homologs, *BrFLS1*, *BrFLS2*, *BrFLS3.1*, *BrFLS3.2*, *BrFLS3.3*, and *BrFLS4.1*, were suggested to be syntenic orthologs of four *AtFLS* genes, *AtFLS1*, *AtFLS2*, *AtFLS3*, and *AtFLS4*, respectively, but those corresponding to *AtFLS5* and *AtFLS6* are absent from the *B. rapa* genome [7]. An additional *FLS* gene (named *BrFLS4.2* in this study) was recently assigned to belong to the FLS4 group, although its syntenic relationship has not been investigated [5]. Therefore, a total of seven *BrFLS* homologous genes have been assigned. Among them, *BrFLS1* (Bra009358), a syntenic ortholog of *AtFLS1*, was predicted to encode a functional FLS enzyme, since *BrFLS1* expression was predominantly higher among other homologs but was not correlated with expression of *BrDFR* genes or anthocyanin accumulation in purple Chinese cabbage [9]. However, the functional differences between the seven *BrFLS* homologs have not been revealed, and the key *FLS* gene mainly responsible for flavonol biosynthesis in *B. rapa* has yet to be determined.

Clustered regularly interspaced short palindromic repeats (CRISPR)/CRISPR-associated (Cas) technology-mediated genome editing (GE) has been used to edit DNA sequences in numerous species due to its simplicity, high efficiency, versatility, and capacity for multiplexing [10]. Since 2015, >45 applications of CRISPR/Cas-mediated GE have been reported in Brassicaceae crops. Most were conducted in *Brassica oleracea* or *B. napus* to improve commercially important agronomic traits or nutritional values such as seed oil, carotenoids, or glucosinolate contents*.* Most of these studies used CRISPR/CRISPR-associated protein 9 (Cas9) to generate insertions/deletions (InDels) in single or multiple genes [11]. Only a few studies have reported modifying genes using CRISPR/Cas9 in *B. rapa*. These studies focused on genes involved in flowering time [12], leaf color transition [13], methylation of pectin [14], self-incompatibility [15], and circadian rhythms [16]. However, considering that the end goal of CRISPR/Cas9 GE is to obtain a transgene-free homozygous plant harboring a precise modification of a specific target gene, and the altered sequence and the resulting traits should be stably inherited, there is still a need for efficiency improvement and diversification of objectives in the CRISPR/Cas9 application for *B. rapa*.

Here, comparative analyses of gene expression and enzyme activities of the *BrFLS* homologs characterized the *BrFLS* family genes and indicated that *BrFLS1* is the only candidate for the major active *FLS* gene in *B. rapa*. The CRISPR/Cas9 targeting *BrFLS1* was introduced to a commercial inbred line of green Chinese cabbage to generate *BrFLS1*-knockout (*brfls1*) plants, and we obtained transgene-free homozygous *brfls1* lines in the T_1_ generation. Flavonol glycosides, the major class of flavonoids in the background variety, dramatically decreased, and instead dihydroflavonol glycosides accumulated in the *brfls1* lines. We showed an effective way of metabolic engineering to develop new varieties of Chinese cabbage with modified flavonoid profiles using CRISPR/Cas9 GE system. Furthermore, our study provides insights into understanding the phenylpropanoid and flavonoid biosynthetic pathway in *B. rapa*.

## Results

### Comparative analysis of gene expression and amino acid sequences of *BrFLS* homologs

**Figure 1:**
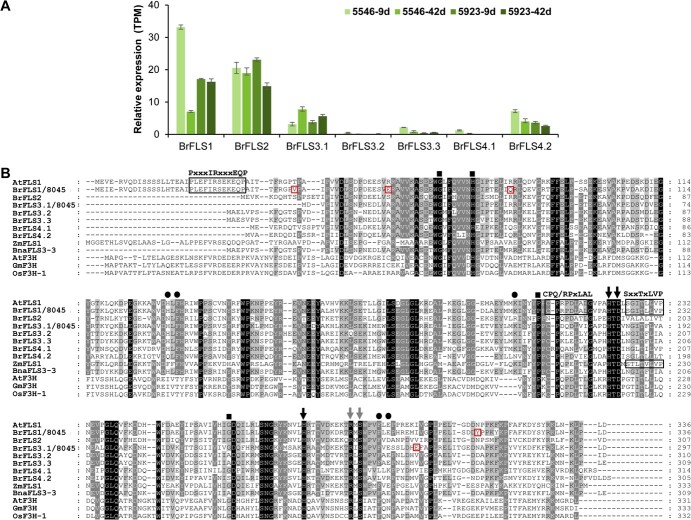
Comparative analysis of gene expression and amino acid sequences of BrFLS homologs. (A) Relative gene expression levels of *BrFLS* homologs based on mRNA-TPM (transcript per million) from the RNA-seq analysis of leaves from 9- and 42-day-old plants of 5546 and 5923 varieties. Error bars indicate ± SD from three replicates. (B) Alignment was conducted using the ClustalW program. Identical amino acids are indicated with black backgrounds. Amino acids that are >80% conserved are indicated with dark grey backgrounds, and those that are >60% conserved are indicated with light grey backgrounds. Three boxes represent FLS-specific motifs ‘PxxxIRxxxEQP’, ‘CPQ/RPxLAL’, and ‘SxxTxLVP’. Amino acid residues responsible for binding ferrous iron and 2-oxoglutarate are marked with black and grey arrows, respectively. Predicted residues involved in substrate binding are marked with black circles. Functional residues suggested to be involved in proper folding of the FLS polypeptide are marked with black squares. Amino acid variations of BrFLS1 and BrFLS3.1 that were cloned from variety 8045 are indicated by red rectangles.

We estimated the expression levels of each *BrFLS* homolog in the leaves of two 9- and 42-day-old green Chinese cabbage varieties, 5546 and 5923, using transcriptome deep sequencing (RNA-seq). Among the seven *BrFLS* genes, *BrFLS1*, *BrFLS2*, *BrFLS3.1*, and *BrFLS4.2* were mainly expressed in all three varieties. In particular, *BrFLS1* and *BrFLS2* showed substantially higher expression levels than the other homologs (Fig. 1A & Supplementary Table S2). The four genes were further investigated for their characterization. According to our pretest, varieties 5546 and 5923 showed very low transformation efficiencies, thus we used another green variety, 8045, which showed high transformation efficiency, as the target variety for further analysis. First, we isolated the CDSs of *BrFLS1*, *BrFLS2*, *BrFLS3.1*, and *BrFLS4.2* from the cauline leaves of variety 8045. Sequencing analysis revealed that the *BrFLS1* CDS differs by 10 nucleotides from the reference sequence (Bra009358) (Supplementary Data Fig. S1), resulting in four amino acid substitutions (T40V, A57E, K83Q, and N313Y) (Fig. 1B), and the *BrFLS3.1* CDS had two nucleotide changes compared to the reference sequence (Bra038648) (Supplementary Data Fig. S2) resulting in one amino acid substitution (K261E) (Fig. 1B), whereas the *BrFLS2* and *BrFLS4.2* coding sequences isolated from the variety 8045 were identical to the reference sequences (Bra038647 and Bra018076, respectively). We aligned the deduced amino acid sequences of the seven BrFLS homologs along with AtFLS1, ZmFLS1 in maize (*Zea mays*), BnaFLS3–3, and three F3Hs including AtF3H, GmF3H in soybean (*Glycine max*), and OsF3H in rice (*Oryza sativa*). All sequences have completely conserved core motifs of 2-ODD, such as ferrous ion binding (HxDxnH) and 2-oxoglutarate binding (RxS) motifs [17], but, in other areas, FLSs and F3Hs were clearly distinguished. Among FLSs, only AtFLS1 and BrFLS1 (BrFLS1/8045) completely share the three FLS-specific ‘PxxxIRxxxEQP’, ‘CPQ/RPxLAL’, and ‘SxxTxLVP’ motifs [18], and ZmFLS1 has the three motifs with partial or complete conservations. BrFLS1 shared 91.4% identity with AtFLS1, whereas the other homologs showed ~60%–67% identity with AtFLS1. These analyses suggest that, among the BrFLS homologs, only BrFLS1 has a complete structure. Notably, BrFLS1 and BrFLS3.1 had conserved residues at the positions proposed to be involved in substrate binding and proper protein folding [19], whereas BrFLS2 and BrFLS4.2 had variations at these positions (Fig. 1B).

### Recombinant BrFLS1 protein is a bifunctional enzyme exhibiting both FLS and F3H activities

**Figure 2:**
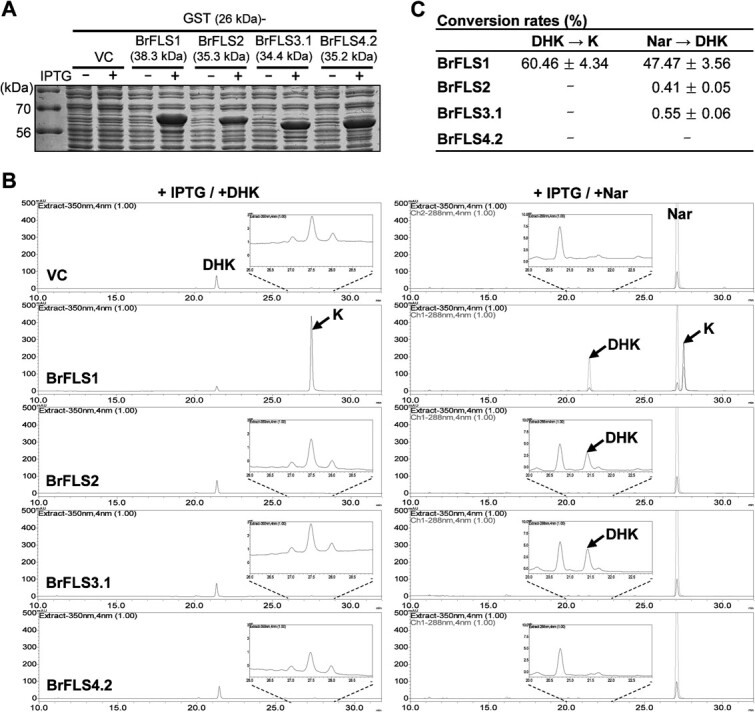
Substrate-feeding assays of recombinant BrFLS homologs. (A) GST-fused BrFLS1, BrFLS2, BrFLS3.1, and BrFLS4.2 were expressed in *E. coli* by IPTG induction, which was verified by SDS-PAGE. VC, vector control. (B) IPTG-induced bacterial cultures were fed with DHK or Nar as a substrate. HPLC identified K production from DHK-fed reactant and DHK and K production from Nar-fed reactant. Nar and DHK were identified at 288 nm (grey chromatogram), and K was identified at 350 nm (black chromatogram). Small peaks of K and DHK are enlarged and shown in the insets. (C) Conversion rates were calculated based on the molar ratio between input and consumed substrates. The mean values were determined from three replicates.

Substrate-feeding assays were performed to characterize the enzymatic properties of recombinant GST-fused BrFLS1, BrFLS2, BrFLS3.1, and BrFLS4.2 with different substrates, DHK or Nar. SDS-PAGE analysis showed the successful production of all recombinant GST-fused proteins upon IPTG induction (Fig. 2A). HPLC analysis of DHK-fed reactants showed that only BrFLS1 produces a large amount of kaempferol (K) from DHK. However, K was almost absent in the reactants of BrFLS2, BrFLS3.1, and BrFLS4.2 (Fig. 2B, left panel). K-like small peaks were shown in the reactants of BrFLS2, BrFLS3.1, and BrFLS4.2, but the same peak at a similar level was detected in the vector control (VC), indicating that these peaks are not specific products by the BrFLSs. Therefore, BrFLS1 was the only enzyme with FLS activity among the four BrFLSs tested. Next, we tested if the BrFLSs also have F3H activity via a Nar feeding as a substrate. We detected DHK and K simultaneously in the Nar-fed reactant of BrFLS1 (Fig. 2B, right panel), indicating that Nar was converted to DHK by the F3H activity of BrFLS1 and then converted to K by its FLS activity, which suggests that BrFLS1 is a bifunctional enzyme with both F3H and FLS activities. At a narrowed range of absorbance, we observed trace levels of DHK in the reactants of BrFLS2 and BrFLS3.1, but not of BrFLS4.2, indicating that BrFLS2 and BrFLS3.1 lack FLS activity but only have faint levels of F3H activity. The conversion rate of each reaction was calculated (Fig. 2C), which showed that BrFLS1 had a conversion rate of 60.5 ± 4.3% for FLS activity and 47.5 ± 3.6% for F3H activity, but the conversion rates of BrFLS2 and BrFLS3.1 for F3H activity were approximately 100-fold lower (0.41 ± 0.05% and 0.55 ± 0.06%, respectively) than that of BrFLS1, suggesting that BrFLS1 is the authentic FLS enzyme with additional F3H activity, and BrFLS2 and BrFLS3.1 can only marginally contribute to dihydroflavonol biosynthesis in *B. rapa*. The bifunctional activity of BrFLS1 was verified by agroinfiltration of *N. benthamiana* leaves (Supplementary Data Fig. S3). Additionally, we supplied the same concentration of DHK or DHQ into the infiltrated leaves and compared the K and quercetin (Q) products. The result showed that K production was ~2.5-fold higher than Q production (Supplementary Data Fig. S4), suggesting a substrate preference biased toward DHK over DHQ.

### Generation of transgene-free homozygous *brfls1* plants

**Figure 3:**
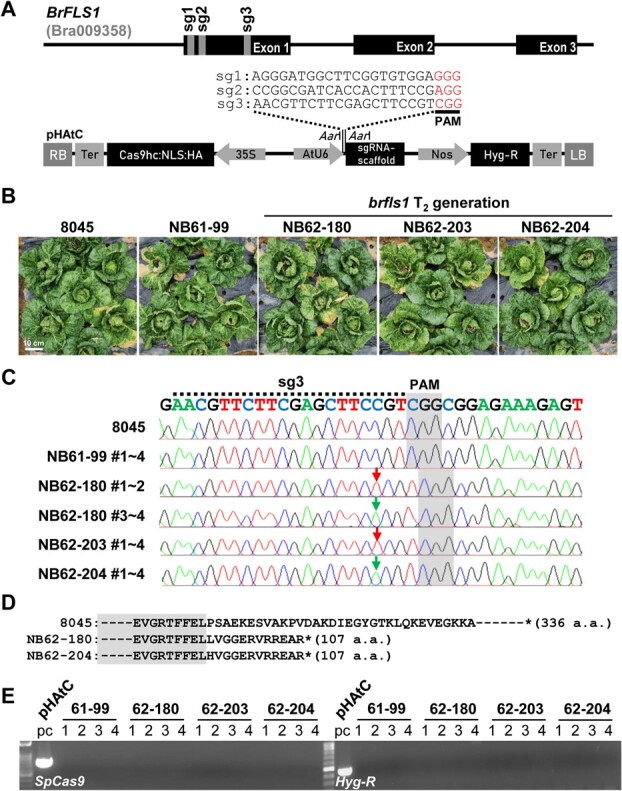
Generation of transgene-free homozygous *brfls1* plants. (A) Diagram of binary vector construction. Three sgRNAs (sg1, sg2, and sg3) were selected in exon 1 of the *BrFLS1* gene and were introduced in the binary vector pHAtC carrying *SpCas9*, sgRNA scaffold, and the hygromycin resistance gene (*Hyg-R*). (B) T_2_ progenies of *brfls1* plants (NB62–180, −203, and −204) and control plants (8045 and T_2_ progenies of NB61–99) grown in green house. (C) The regions encompassing the sg3 target site (dashed line) were amplified by genomic PCR and sequenced, which showed single T (red arrow) or A (green arrow) insertion 3 bp upstream of the PAM sequence (5′-CGG-3′) (shaded grey) in the target site of *BrFLS1* in the *brfls1* T_2_ plants. (D) Deduced amino acid sequences of the *brfls1* harboring the T or A insertion show early stops of BrFLS1 translation after 107^th^ amino acid caused by a frameshift. (E) Genomic PCR of four individuals (1–4) of the *brfls1* T_2_ progenies showing absence of transgene in their genome.

We generated *BrFLS1*-knockout Chinese cabbage using CRISPR/Cas9. To induce an InDel mutation leading to a frameshift in the *BrFLS1* coding region, we designed three sgRNAs (sg1, sg2, and sg3) targeting specific sequences on the first exon of *BrFLS1.* Each sgRNA was introduced into the binary vector pHAtC harboring a 35S promoter-driven *Streptococcus pyogenes Cas9* (*SpCas9*) expression cassette (Fig. 3A) [20] and applied for *Agrobacterium*-mediated transformation of variety 8045. We obtained four T_0_ plants (NB60, 61, 62, and NB63) from the sg3-transformed calli. The transformation efficiency was ~1.2% (four T_0_ plants/336 explants). Targeted deep-sequencing analysis revealed that the T_0_ plants that harbored single A or T insertions mainly occurred 3 bp upstream of the PAM sequence in their *BrFLS1* sequences. The editing efficiencies of T_0_ plants were 81.6% in NB60, 11.3% in NB61, 99.8% in NB62, and 0.2% in NB63. We tried to get transgene-free homozygous lines in the T_1_ generation. We obtained the transgene-free T_1_ plants in NB61 and NB62 with 58.7% and 3.5% probabilities, respectively. However, we were able to get transgene-free homozygous *BrFLS1*-knockout T_1_ plants from only NB62 (NB62–180, −203, and − 204), and they were referred to as *brfls1* (Supplementary Table S3). We performed off-target analyses on four predicted off-target sequences similar to the sg3 target sites and the corresponding sequences in the six other *BrFLS* homologs, which identified no mutations in the sequences in the three *brfls1* T_1_ plants (Supplementary Table S4). T_2_ progenies of the *brfls1* plants were generated and cultivated in the greenhouse to form head together with the variety 8045 and non-edited T_2_ progenies of NB61–99 as controls (Fig. 3B). Genomic PCR and sequencing analysis verified that the A or T insertion in the target site resulting in the C-terminal truncation of BrFLS1 (Fig 3C and3D) and the absence of the transgene (Fig 3E) were successfully inherited in the *brfls1* T_2_ progenies.

### Significant reduction of flavonols and concomitant accumulation of dihydroflavonols in the *brfls1* T_2_ plants

**Figure 4:**
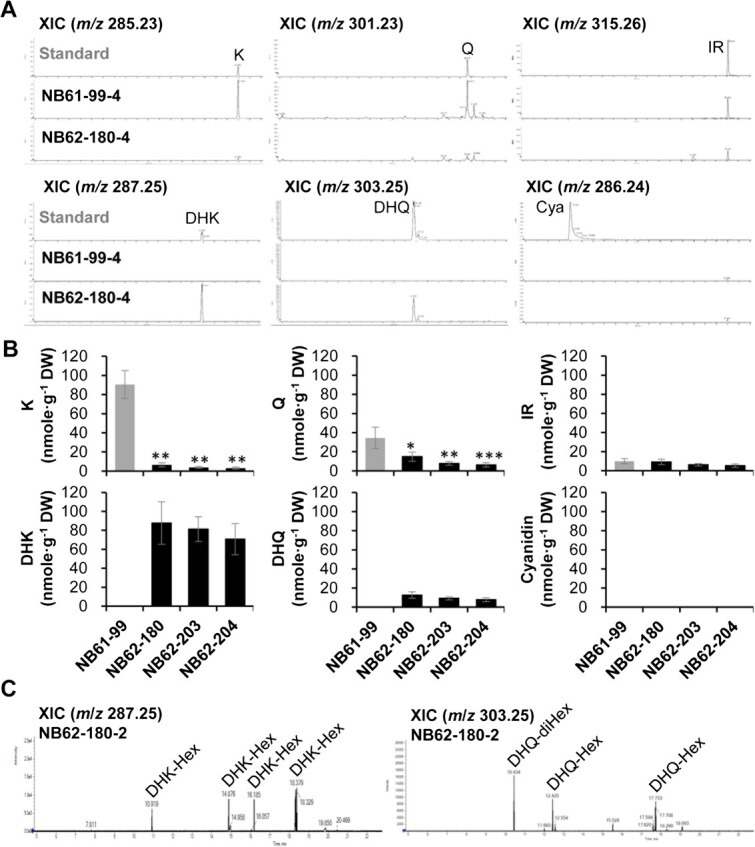
Investigation of changes in the flavonoid profile of *brfls1* plants using LC-ESI-QTOF-MS analysis. LC-ESI-QTOF-MS analysis of flavonoid aglycones extracted from the leaves of four T_2_ individuals generated from the NB61–99 (WT) and *brfls1* (NB62–180, NB62–203, NB62–204) with negative ionization mode. (A) Representative XICs displaying mass spectra of flavonoid aglycones in the T_2_ plants. XICs at *m/z* 285.23, *m/z* 287.25, *m/z* 301.23, *m/z* 303.25, *m/z* 315.26, and *m/z* 286.24 show deprotonated K, DHK, Q, DHQ, IR, and cyanidin Cya aglycones, respectively. (B) The average values of flavonoid content in the four individuals of each T_2_ line were calculated based on the areas of corresponding standards. The mean values ± SD of four independent biological samples are shown. Significant differences were determined by Student’s *t*-tests. Asterisks indicate significant differences from the WT (^*^P < 0.05, ^**^P < 0.01, ^***^P < 0.001). (C) Representative XICs of dihydroflavonol glycosides extracted from the *brfls1* T_2_ plants using 70% methanol (Hex, hexoside; diHex, dihexoside).

To investigate whether the flavonoid content and profile changed, we sampled leaves from the middle layers of the cabbage head from four *brfls1* T_2_ individuals of each line and performed liquid chromatography and electrospray ionization quadrupole time-of-flight mass spectrometry (LC-ESI-QTOF-MS) with negative ionization mode. Flavonoid aglycones were extracted from the lyophilized leaves with acid hydrolysis and analyzed along with K, Q, DHK, and DHQ aglycones as standards. The extracted ion chromatograms (XICs) showed significant reduction of K (*m/z* 285.23 [M-H]^−^) and Q (*m/z* 301.23 [M-H]^−^) in the *brfls1* lines. Approximately 93%–97% (84–87 nmole·g^−1^ FW) of K and 57%–82% (20–28 nmole·g^−1^ FW) of Q were reduced in the *brfls1* T_2_ progenies compared to the NB61–99 progenies. On the other hand, DHK (*m/z* 287.25 [M-H]^−^) and DHQ (*m/z* 303.25 [M-H]^−^) that are absent in the NB61–99 progenies accumulated in the *brfls1* T_2_ progenies (Fig. 4A) to ~71–88 nmole·g^−1^ FW and 8–13 nmole·g^−1^ FW, respectively, which are comparable to the reduced levels of K and Q. However, isorhamnetin (IR: *m/z* 315.26 [M-H]^−^) contents were not significantly changed and cyanidin (Cya: *m/z* 286.24 [M-H]^−^) was absent in the *brfls1* plants. These results indicate that blocking flavonol biosynthesis resulted in accumulation of its dihydroflavonol precursors in green Chinese cabbage. Furthermore, LC-ESI-QTOF-MS/MS analysis of 70% (v/v) methanol extracts of the leaves identified dihydroflavnol glycosides accumulated in the brfls1, which showed four and three major peaks corresponding to DHK and DHQ glycosides, respectively, in the *brfls1* T_2_ plants. According to their MS/MS spectra, they were tentatively assigned as three different DHK-hexosides and one DHQ-dihexoside and two different DHQ-hexosides, respectively (Fig. 4C and Supplementary Data Fig. S5). Although the presence of other minor contributors in flavonol biosynthesis cannot be ruled out, these results clearly show that BrFLS1 is the major active FLS enzyme in Chinese cabbage.

### Upregulation of the phenylpropanoid and flavonoid biosynthetic genes caused slight increases in total phenolics in the *brfls1* T_2_ plants

**Figure 5:**
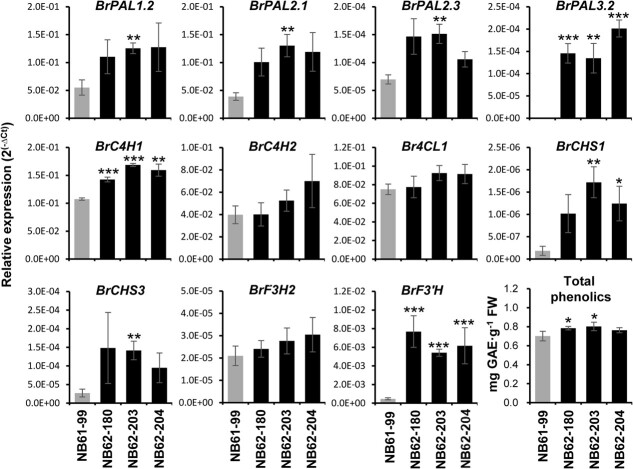
Analyses of changes in the expression of flavonoid biosynthetic genes and total phenolic contents in the *brfls1* T_2_ plants. qPCR analysis was performed and total phenolic content was measured in the four T_2_ individuals generated from the NB61–99 and *brfls1* (NB62–180, NB62–203, NB62–204). Expression values were normalized using *B. rapa Actin7* (*BrACT7*) transcript. The total phenolic contents were calculated using gallic acid as a standard, and the mean values were expressed as milligrams of gallic acid equivalents per gram of fresh weight (mg GAE·g^−1^ FW). The mean values ± SD of four independent biological samples are shown. Significant differences were determined by Student’s *t*-tests. Asterisks indicate significant differences from the WT (^*^P < 0.05, ^**^P < 0.01, ^***^P < 0.001).

To explore whether the change in the flavonoid profile caused by knocking out *BrFLS1* was accompanied by changes in the expression patterns of phenylpropanoid and flavonoid biosynthetic genes, we performed qPCR analysis with total RNA isolated from the T_2_ plants of *brfls1* and NB61–99. The early step of phenylpropanoid and flavonoid biosynthesis, such as *BrPAL*s (*BrPAL1.2*, *BrPAL2.1*, *BrPAL2.3*, and *BrPAL3.2*), *BrC4H1* and *BrCHS*s (*BrCHS1* and *BrCHS3*), and *BrF3′H* that is responsible for flavonoid B-ring hydroxylation were remarkably upregulated in the *brfls1* plants. These results suggest that metabolic flux into the lignin branch or flavonoid branch can be enhanced by blocking *BrFLS1*. Thus, we examined whether levels of total phenolics were changed in the *brfls1* plants, and we found that the total phenolic contents slightly but significantly increased to ~13% in the *brfls1* plants compared to the control plants (Fig. 5).

### Phenotypic changes of the *brfls1* plants

**Figure 6:**
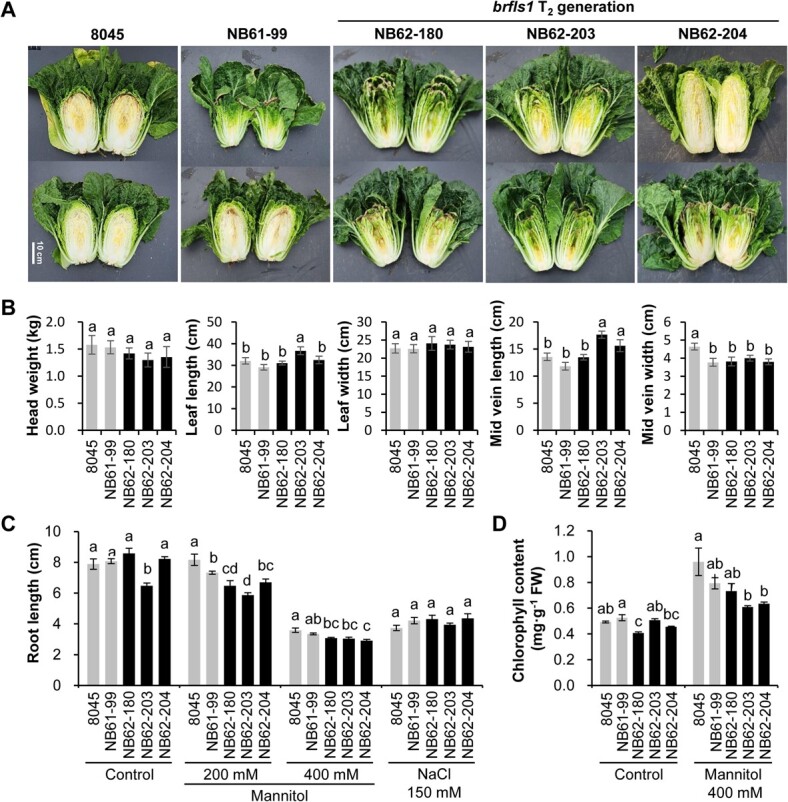
Phenotypic analyses of the *brfls1* T_2_ plants. (A) Cross-sections of heads of two *brfls1* T_2_ plants and controls (8045 and NB61–99). (B) Indices related to agricultural traits of their heads (C) Root lengths of seedlings of *brfls1* T_2_ plants and control plants four (for control treatment) or six (for mannitol or NaCl treatment) days after osmolytes treatment. (D) Chlorophyll contents measured from aerial parts of the seedlings 4 days after mannitol treatment. The mean values ± SD of four independent biological samples are shown. Statistical significance was determined by Duncan’s Multiple Range Test using SAS (version 9.1) software. Significant differences between means (*P* < 0.05) are indicated by different lower case letters (a, b, and c).

We surveyed the agronomic traits of *brfls1* T_2_ Chinese cabbage, indicating that their heads were somewhat lighter than the controls, and although there were individual variations, tip burn symptoms were observed in the middle layers of the *brfls1* heads. Their shape tended to be vertically elongated due to the increased mid-vein lengths compared to the controls (Fig. 6A and6B). In terms of reproductive phenotypes, there were no significant differences in bolting, flowering, seed color, and seed weight between *brfls1* and the controls (Supplementary Data Fig. S6). However, root lengths and chlorophyll contents of *brfls1* seedlings were reduced slightly but significantly by a plethora of mannitol (200 mM or 400 mM), but not of NaCl (150 m), compared to those of the controls (Fig. 6C,6D, Supplementary Data Fig. S7, andS8), suggesting that flavonols play a positive role in osmotic stress tolerance during early growth of Chinese cabbage.

## Discussion

In the Brassicaceae lineage, integrative studies on *FLS* family genes were previously carried out in *A. thaliana* [3, 18] and *B. napus* [5], providing important references for understanding the characteristics of the *BrFLS* family. The expression of *FLS1*s, *FLS2*s, and *FLS3*s was commonly observed in *A. thaliana, B. napus*, and *B. rapa*; however, the expression patterns of each group are species-specific. *FLS1*s are predominantly expressed in all three species, whereas *FLS2*s are expressed at high levels in *A. thaliana* and *B. rapa* but at marginal levels in *B. napus*. *FLS3*s are expressed at minor levels in all three species except for *BnaFLS3–3* and *BnaFLS3–4*, which are highly expressed in *B. napus*. Interestingly, nonfunctional *AtFLS2*, a pseudogene encoding a protein lacking the key C-terminal [18], was inherited as a functional BrFLS2 exhibiting F3H activity *in B. rapa*. Furthermore, the BrFLS2 was inherited in *B. napus* as nonfunctional *BnaFLS2*s pseudogenized again [5]. In the case of the FLS3s, AtFLS3, exhibiting only marginal FLS activity, was inherited in *B. rapa* as BrFLS3.1 exhibiting F3H activity, but afterward, BrFLS3.1 has been inherited in *B. napus* as BnaFLS3–1 and BnaFLS3–2 almost pseudogenized. Moreover, the BnaFLS3–3 and BnaFLS3–4 exhibiting F3H activity were derived from BrFLS3–2 and BrFLS3–3, which were shown not to be expressed (seemed to be almost pseudogenized) in this study. The dynamic history of Brassicaceae *FLS* evolution implies that flavonoid biosynthesis has been enhanced during evolution of *Brassica* species.

Comparing the deduced amino acid sequences of the seven BrFLSs simply shows that only *BrFLS1* encodes complete structure for authentic FLS in the *B. rapa* genome. The substrate-feeding assays of BrFLS1 showed that its F3H and FLS activities were nearly comparable, suggesting that BrFLS1 contributes to dihydroflavonol production at a substantial level along with BrF3H in Chinese cabbage. Even though the amino acid sequences of BrFLS2 and BrFLS3.1 are distinct from those of BrF3Hs, they were shown to have only F3H activity but not FLS activity (Fig. 2) like BnaFLS3.3 and BnaFLS3.4 [5], which indicates that the ‘PxxxIPxxxEQP’ motif is unnecessary for their F3H activities. However, considering that Maize (*Z. mays*) ZmFLS1, exhibiting only FLS activity [21], has partially matching ‘PxxxIPxxxEQP’ and ‘CPQ/RPxLAL’ motifs and a completely conserved SxxTxLVP motif, the existence of the three motifs itself is likely to be essential for FLS activity, regardless of whether it is FLS mono- or FLS/F3H bifunctional enzyme, and it can be further assumed that modest changes in these motifs can result in loss of F3H activity. The transient expression of *BrFLS1* in *N. benthamiana* leaves showed a substrate preference of BrFLS1 biased towards DHK rather than DHQ. Likewise, AtFLS1 prefers DHK to DHQ as a substrate, and its His-132 was proposed to be the residue responsible for the substrate preference [19]. BrFLS1 also has an His-132 residue, which may account for its substrate preference for DHK.

Flavonoid analysis showed drastic decreases in flavonol glycosides and parallel accumulation of dihydroflavonol glycosides in the *brfls1* plants. To our knowledge, the accumulation of dihydroflavonol glycosides is the first reported in *B. rapa*. However, similar to the *A. thaliana FLS1* mutant (*atfls1*) [3], we observed residual flavonols in the *brfls1* plants. Considering our results of the sequence analysis and substrate-feeding assays, the source of the residual FLS activity was unlikely to come from the other BrFLSs. AtANS has been known as an alternative route for flavonol production in *A. thaliana*, and the *atans atfls1* double mutant lacked most of the residual flavonols in *A. thaliana* [18], which supports the possibility that BrANS is a minor contributor in flavonol production in Chinese cabbage, although the expression levels of *BrANS*s in the *brfls1*, a green variety, is presumed to be very low [9]. The *atfls1* mutant shows drastic reduction of flavonol glycosides and parallel accumulation of anthocyanins as well as dihydroflavonol glycosides, which has been suggested to be due to activation of the late flavonoid biosynthesis genes (LBGs), e.g. *DFR*, *ANS*, and *UDP-glucose:flavonoid 3-O-glucosyltransferases* (*UGT*s), and *acyltransferases* (*AT*s) in association with flavonol-responsive regulators [18]. Unlike *atfls1*, the *brfls1* plants did not accumulate anthocyanins, which can be explained by the previous finding that green Chinese cabbages carry a defective *BrMYB2* allele containing a suppressive long intron, that makes green Chinese cabbage unable to produce anthocyanins [22]. The levels of decreased flavonols were nearly equivalent to the levels of accumulated dihydroflavonols, implying that most of the metabolic flux converged into dihydroflavonols without leakage to the anthocyanin branch in the *brfls1* plants. Hence, if we focus on the special trait of Chinese cabbage accumulating dihydroflavonols, the genetic characteristics of green Chinese cabbage can be considered as a great advantage.

*BrPALs, BrC4H, BrCHSs,* and *BrF3′H* were upregulated in the *brfls1* plants, which suggests a possibility that flavonol itself can be involved in regulating the phenylpropanoid and flavonoid biosynthetic pathway in *B. rapa*. Similar patterns were reported in *atfls1*, in which *AtPAL1, AtPAL2, AtDFR*, and *AtANS* were upregulated [18, 23]*.* Importantly, flavonol aglycones, rising from compromised flavonol-3-*O*-conjugation in the *ugt78d1*/*ugt78d2* double mutant, led to feedback inhibition of phenylpropanoid and flavonoid biosynthetic genes, but the inhibitions were released in the *atchs ugt78d1 ugt78d2* triple mutant in which flavonol production is completely blocked [23], which provides strong evidence that flavonol aglycones are important regulatory compounds in the phenylpropanoid and flavonoid biosynthetic pathway. Therefore, it can be speculated that the gene expression of *BrPALs*, *BrC4H*, *BrCHSs*, and *BrF3′H* in wild type is weakly inhibited by trace levels of flavonol aglycones, but in the *brfls1*, the opportunity for nascent free aglycones to be released is deprived by the drastic reduction of flavonol production and the resulting relatively excess capacity of UGT activity. Thereby, the inhibition at basal levels is released, resulting in upregulation of the genes in the *brfls1* plants. Indeed, LC-ESI-QTOF-MS analysis of NB61–99 showed trace amounts of flavonol aglycones (Supplementary Data Fig. S9).

The change in flavonoid profile seemed to negatively affect early growth of *brfls1*, suggesting the positive effect of flavonols on abiotic stress tolerance in Chinese cabbage. However, despite its vulnerability in stress at the seedling stage, it did not cause any disruption to growth under normal condition. As a result, the overall agricultural traits of *brfls1* Chinese cabbages, such as head formation and proliferation, are within the normal range, showing that their agricultural utility value is sufficient. But one thing to consider is whether abandoning the positive aspects of flavonols and replacing them with dihydroflavonols would have agricultural and dietary benefits. Flavonols are involved in various aspects of plant physiology as well as human health mainly due to their antioxidant properties. However, from an agricultural perspective, excessive accumulation of flavonols can detract from the commercial value of agricultural products. Insoluble flavonols may impair digestibility [24], and a kaempferol-glycoside causes the bitter taste of rapeseed (*B. napus* subsp. *napus*) protein isolate [25]. Accordingly, yellow-seeded *B. napus* varieties with reduced phenolics, e.g. epicatechin, isorhamnetin glucoside, and kaempferol glucosides, have been developed through interspecific hybridization ( [26]; J. [27]). Therefore, the value of the *brfls1* plants needs to be considered in terms of dietary properties. The accumulation of dihydroflavonols is also of interest as various pharmaceutical properties of dihydroflavonols have been reported, such as anti-inflammation [28], improvement of insulin resistance [29], promotion of apoptosis [30], prevention of Alzheimer’s disease [31], inhibition of T-cell activation [32], and protection of neuronal cells [33]. Although it is not clear to what extent these functionalities of dihydroflavonols are superior to those of flavonols, the *brfls1* Chinese cabbage lines could be considered as unique breeding resources to develop functional foods with improved bioactive or pharmaceutical properties.

## Materials and methods

### Plant materials and growth conditions

Seeds of three inbred lines of green Chinese cabbage (varieties 5546, 5923, and 8045) were provided by Asiaseed Inc. (Icheon, South Korea). Nine-day-old seedlings, grown on Murashige and Skoog (MS) medium, were further grown in soil for 42 days under 16-h/8-h light/dark condition at 25°C. Cauline leaves of the 9- and 42-day-old plants were harvested for total RNA extraction. To induce flowering and silique formation, young seedlings grown on soil for ~2 weeks were vernalized for ~6 weeks.

### Total RNA extraction and RNA sequencing analysis

Cauline leaves of the 9- and 42-day-old plants were used for total RNA extraction using a FavorPrep Plant Total RNA Mini Kit (Favorgen, Pingtung, Taiwan, China). RNA samples were sent to Theragen Bio Corp. (Gyeonggi Province, Republic of Korea) for RNA sequencing with an Illumina HiSeq 4000 system (Illumina, San Diego, CA, USA). The sequence quality of raw reads was evaluated with FastQC (version 0.11.9). High-quality reads were aligned to reference genome of Chinese cabbage (http://brassicadb.org/brad/) using TopHat2 (version 2.1.1) [34]. Expression levels of genes were quantified with Cufflinks protocol [35], and transcript per million (TPM) values were used to determine expression levels.

### Cloning and bacterial expression of *BrFLS* genes

The coding sequences (CDSs) of *BrFLS*s were isolated from the cauline leaves of green Chinese cabbage by reverse transcription-PCR using anfiRivert cDNA Synthesis Master mix (GenDEPOT, Barker, TX, USA), Q5 High-Fidelity DNA polymerase (New England Biolabs, Ipswich, MA, USA), and gene-specific primers (Supplementary Table S1). The CDSs were cloned into the pGEX-6P-1 vector and verified by sequencing, followed by their transformation into *Escherichia coli* BL21 (DE3) (Novagen, Darmstadt, Germany).

### Substrate-feeding assay of recombinant BrFLS proteins

Recombinant glutathione S-transferase (GST)-fused BrFLS proteins expression was induced by 100 μM isopropyl β-D-1-thiogalactopyranoside (IPTG) at 20°C for 4 h. Small aliquots of the induced cultures were set aside for SDS-PAGE analysis. A total of 200 μM of (±)-dihydrokaempferol (DHK) or (±)-naringenin (Nar) (Sigma-Aldrich, St. Louis, MO, USA) was added to the induced culture medium as a substrate. After 2 h incubation at 25°C, the culture medium was extracted with the same volume of ethyl acetate. The upper ethyl acetate phase was evaporated, and the resulting residue was recovered by dissolving in methanol and analyzed by high-performance liquid chromatography (HPLC) with a protocol previously described [36], The production of protein was verified by SDS-PAGE.

### Transient expression of *BrFLS1* in *Nicotiana benthamiana* leaves

*BrFLS1* CDS was introduced into the binary vector pEarlyGate201 [37] via the Gateway cloning system to create a fusion construct containing N-terminal HA tag. Transformation of Agrobacterium GV3101 with the binary vector was carried out with the freeze–thaw method [38]. The transformed Agrobacterium was initially infiltrated into *N. benthamiana* leaves according to the previous method [36], and 4 days later, 100 μM of (±)-Nar, (±)-DHK, or (±)-dihydroquercetin (DHQ) (Sigma-Aldrich) was infiltrated into the same leaves as a substrate, followed by incubation for 24 h. The leaf samples were extracted with 2 N HCl-containing 50% (v/v) methanol at 95°C for 2 h to convert flavonoid glycosides to aglycones and then analyzed by HPLC.

### Western blot analysis

Total protein of *N. benthamiana* leaves were extracted with an appropriate buffer (50 mM Tris-Cl (pH 8.0), 250 mM sucrose, 2 mM EDTA, 2 mM DTT, and 200 μM phenylmethylsulfonyl fluoride). The supernatant separated through centrifugation (10 000 × g for 10 min) was used as a total protein, of which 20 μg was applied for Western blot analysis with the anti-HA antibody according to the previous mehod [39].

### Construction of the CRISPR/Cas9 binary vector

Cas-Designer, a web-based guide RNA designing tool (http://rgenome.net) [40] was used to identify potential single-guide RNA (sgRNA) sequences based on the genomic sequence of *BrFLS1*. Among them, three sequences (sg1, 5′-AGGGATGGCTTCGGTGTGGA-3′; sg2, 5′- CCGGCGATCACCACTTTCCG-3′; sg3, 5′-AACGTTCTTCGAGCTTCCGT-3′) next to the protospacer adjacent motif (PAM) sequences (5′-NGG-3′) located within the first exon were selected, with expected out-of-frame scores of 68%, 60%, and 63%, respectively. DNA oligonucleotides of each sgRNA sequence were synthesized in the forward and reverse orientations and annealed by decreasing the temperature from 95°C to 25°C for 1.5 h. The resulting sgRNA DNA fragments were cloned into the binary vector pHAtC, according to a previously reported protocol [20]. The resulting CRISPR/Cas9 binary vector was introduced into Agrobacterium strain GV3101 by the freeze–thaw method.

### Chinese cabbage transformation

The Chinese cabbage transformation method was described previously [16]. The newly formed T_0_ plants were transferred to soil after acclimatization and grown in a glasshouse for 4–5 days, vernalized at 4°C for ~45 days, and returned to the glasshouse. Genomic DNA of the cauline leaves from each T_0_ plant was isolated and genotyped with PCR to detect the hygromycin resistance and *SpCas9* genes. After flowering, bud pollination of T_0_ plant was conducted to obtain T_1_ seeds. Genotyping of T_1_ lines was conducted by PCR, and individual plants lacking the hygromycin resistance and *SpCas9* genes were selected and further analyzed by PCR and sequencing of the target site to verify editing of *BrFLS1*. The primers used for genotyping are listed in Supplementary Table S1.

### Off-target mutation analysis

Potential off-target edits of the sgRNAs were identified in the *B. rapa* genome using the CRISPR-GE web tool (http://ski.scau.edu.cn) [41]. BLAST hits containing potential off-target sequences that had fewer than four mismatches and were located in the coding sequence were selected (Supplementary Table S2). The sequences of other *BrFLS* genes corresponding to the target site were also considered as potential off-targets. PCR using specific primers (Supplementary Table S1) was performed to amplify the potential off-target sequences in the T_1_ lines, and the products were analyzed by sequencing.

### Identification of flavonoid aglycones and glycosides

The leaf parts of the middle layers forming head of 75-day-old Chinese cabbages were sampled and lyophilized. Ground powder of the sample was extracted with 2 N HCl-containing 50% methanol at 95°C for 2 h for acid hydrolysis of flavonoids or with 70% (v/v) methanol for 30 min at 70°C for flavonoid glycosides extraction. The extracts were injected into a Shimadzu liquid chromatography (LC) system (Nexera X2 UHPLC) (Shimadzu) connected to a reversed-phase Shim-pack GIS-ODS-I column (3 μm, 3.0 × 100 mm) (Shimadzu). Water containing 0.1% (v/v) formic acid (Sol. A) and acetonitrile containing 0.1% (v/v) formic acid (Sol. B) were used as eluents with a flow rate of 0.5 ml·min^−1^. The temperature of column oven was maintained at 35°C, and the elution condition was optimized as follows: 0–1 min, 10% Sol. B; 1–25 min, 10%–100% Sol. B; 25–40 min, 100% Sol. B; 40–41 min, 10% sol. B (all v/v). Flavonoid aglycones and glycosides were analyzed by a quadrupole time-of-flight mass spectrometer (QTOF-MS) (TripleTOF™ 5600^+^, AB Sciex, Ontario, CA, USA) in electrospray ionization (ESI) modes. Peak extraction was performed by integration software (MasterView v1.1; AB Sciex). Mass spectrometry conditions were optimized as follows: curtain gas, 25 psi; heating gas, 50 psi; nebulizing gas, 50 psi; ion spray voltage, floating between 4.5 and 5.5 kV; temperature, 550°C; fragmentation, 35 collision energy and 15 collision energy spread; scan range, 50–1500 m/z. K, Q, (±)-DHK, (±)-DHQ, IR, and Cya (Sigma-Aldrich) were used as standards for identification and quantification of flavonoid aglycones in samples.

### Total phenolic content measurement

The leaf part of the middle layer of 75-day-old Chinese cabbage was extracted with 80% methanol for 24 h at room temperature in darkness. The supernatant separated by centrifugation was mixed with the same volume of 0.5 N Folin & Ciocalteu’s phenol reagent. After 5 min of incubation, 2.8 volume of 20% (w/v) Na_2_CO_3_ was added to the mixture and incubated in darkness for 2 h. Absorbance of the mixture was measured at 760 nm, and the total phenolic content of the sample was calculated using gallic acid as a standard. The result was expressed in milligrams of gallic acid equivalents per gram of fresh weight (mg GAE·g^−1^ FW).

### Quantitative PCR

Total RNAs were isolated from the leaf part of the middle layer of 75-day-old Chinese cabbage, and first-strand cDNA was synthesized as described above. The cDNA samples were diluted 3-fold and used for quantitative PCR (qPCR). The qPCR was conducted with AccuPower 2X GreenStar qPCR Master Mix (Bioneer, Daejun, Republic of Korea) and primer sets (Supplementary Table S1). The PCR was performed with Bio-Rad CFX96 system (Bio-Rad Laboratories, CA, USA) under the following condition: 95°C for 5 min; 50 cycles at 95°C for 15 s; 58°C for 30 s. Reactions were carried out in triplicate, and data were normalized using *B. rapa Actin7* (*BrACT7*) as a housekeeping gene (Supplementary Table S1).

### Osmolytes treatment of Chinese cabbage

Nine-day-old seedlings of Chinese cabbage grown on solid MS medium containing 1% sucrose were transferred to the solid MS medium containing mannitol (200 mM or 400 μM) or NaCl (150 mM). After incubation for 6 days, their root lengths were measured, and their aerial parts were detached from the roots for chlorophyll content measurement with DMSO method described previously [42].

## Acknowledgements

This work was supported by the New Breeding Technologies Development Program [grant number PJ016545] of the Rural Development Administration, Republic of Korea.

## Author contributions

S.P. performed gene cloning, enzyme assay, HPLC, LC-ESI-QTOF-MS analysis, and wrote the manuscript. H.L. performed gene cloning, HPLC analysis, Chinese cabbage breeding, and off-target analysis. J.H., J.S., C.J.L., and J.O. contributed to the Chinese cabbage breeding, cultivation, and genotyping. S.H.L. analyzed the LC-ESI-QTOF-MS data. S.B.L., J.L., and S.L. performed data analysis and review. J.A.K. was in charge of Chinese cabbage transformation. B.K. designed and supervised this research and revised the final version of the manuscript.

## Data availability

All relevant data generated or analyzed are included in the manuscript or in supplementary materials.

## Conflict of interest statement

No conflict of interest declared.

## Supplementary Data

Supplementary data is available at *Horticulture Research* online.

## Supplementary Material

Web_Material_uhad239
