# Supplementary material for: Gene editing of authentic Brassica rapa flavonol synthase 1 generates dihydroflavonol-accumulating Chinese cabbage
Source: Hortic Res. 2023 Nov 14;10(12):uhad239. doi: 10.1093/hr/uhad239 (PMC10716633; doi:10.1093/hr/uhad239)
Supplement: Web_Material_uhad239 [file web_material_uhad239.docx]

**Supplementary Data**

**
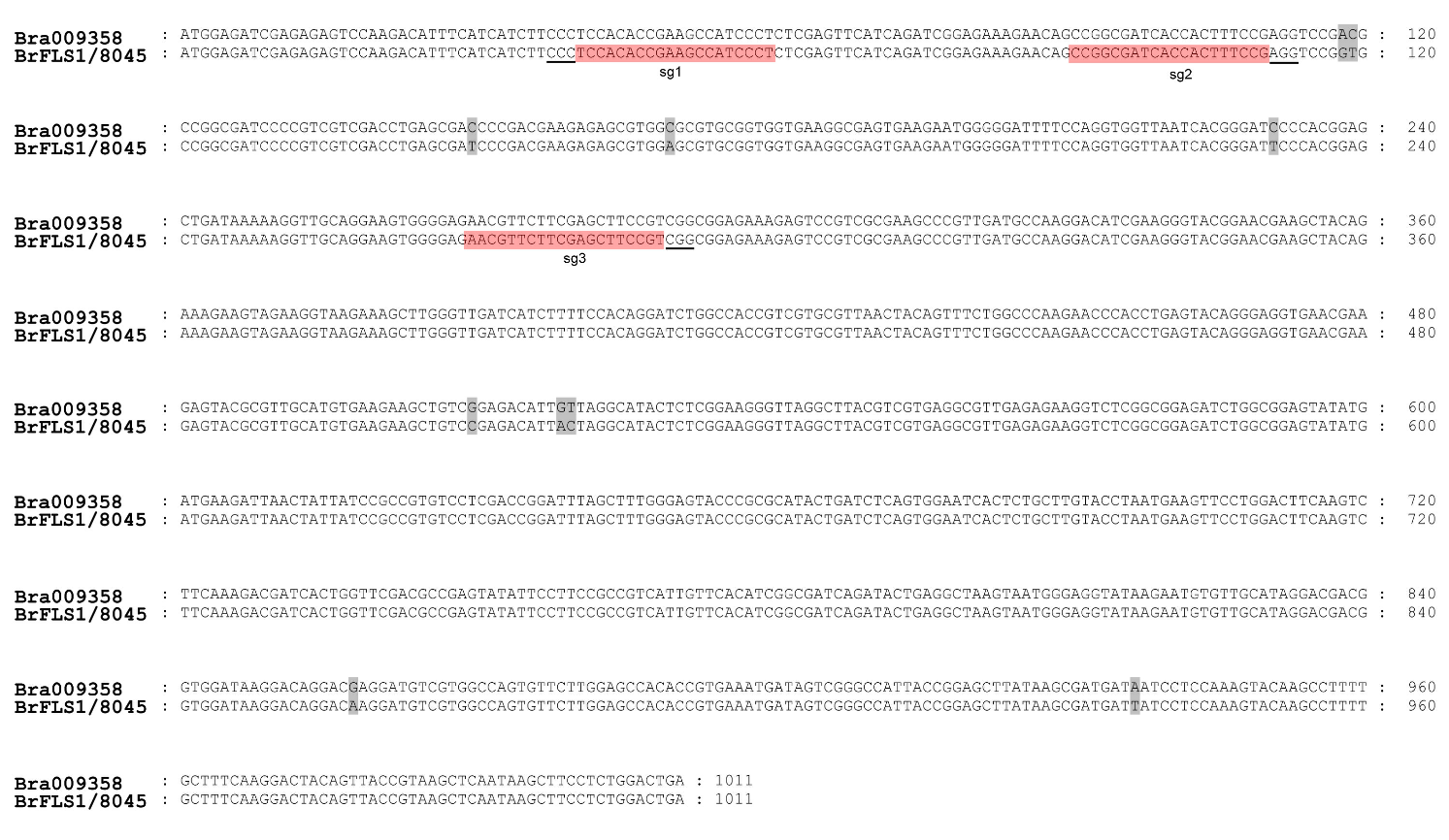
**

**Figure S1. Comparison of *BrFLS1* coding sequences between registered sequence (Bra009358) and the sequence cloned from variety 8045.**

Different nucleotides are indicated by grey boxes. sgRNA sequences (sg1, sg2, and sg3) and adjacent PAM sequences are indicated by red box and are underlined, respectively.


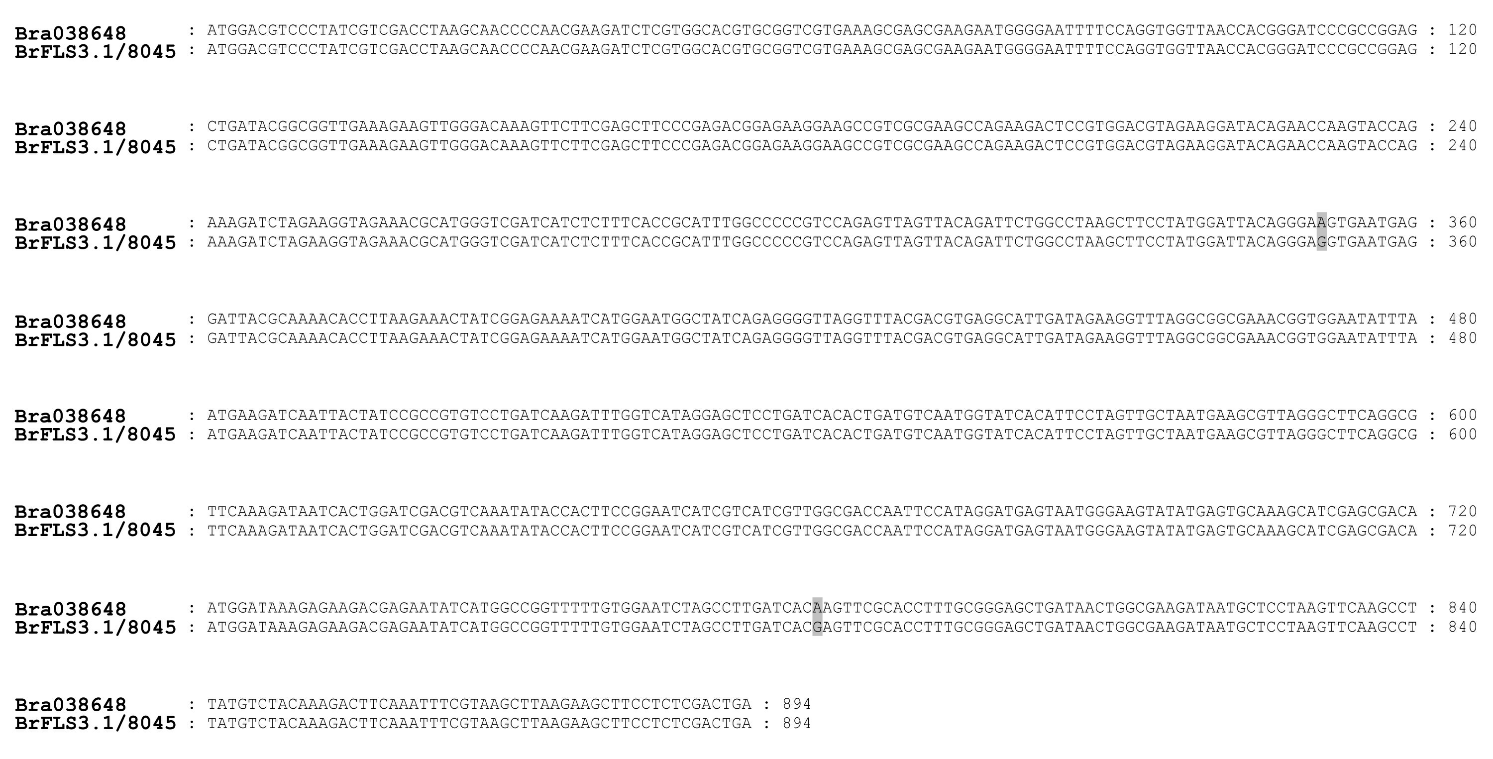


**Figure S2. Comparison of *BrFLS3.1* coding sequences between registered sequence (Bra038648) and the sequence cloned from variety 8045.**

Different nucleotides are indicated by grey boxes.

**
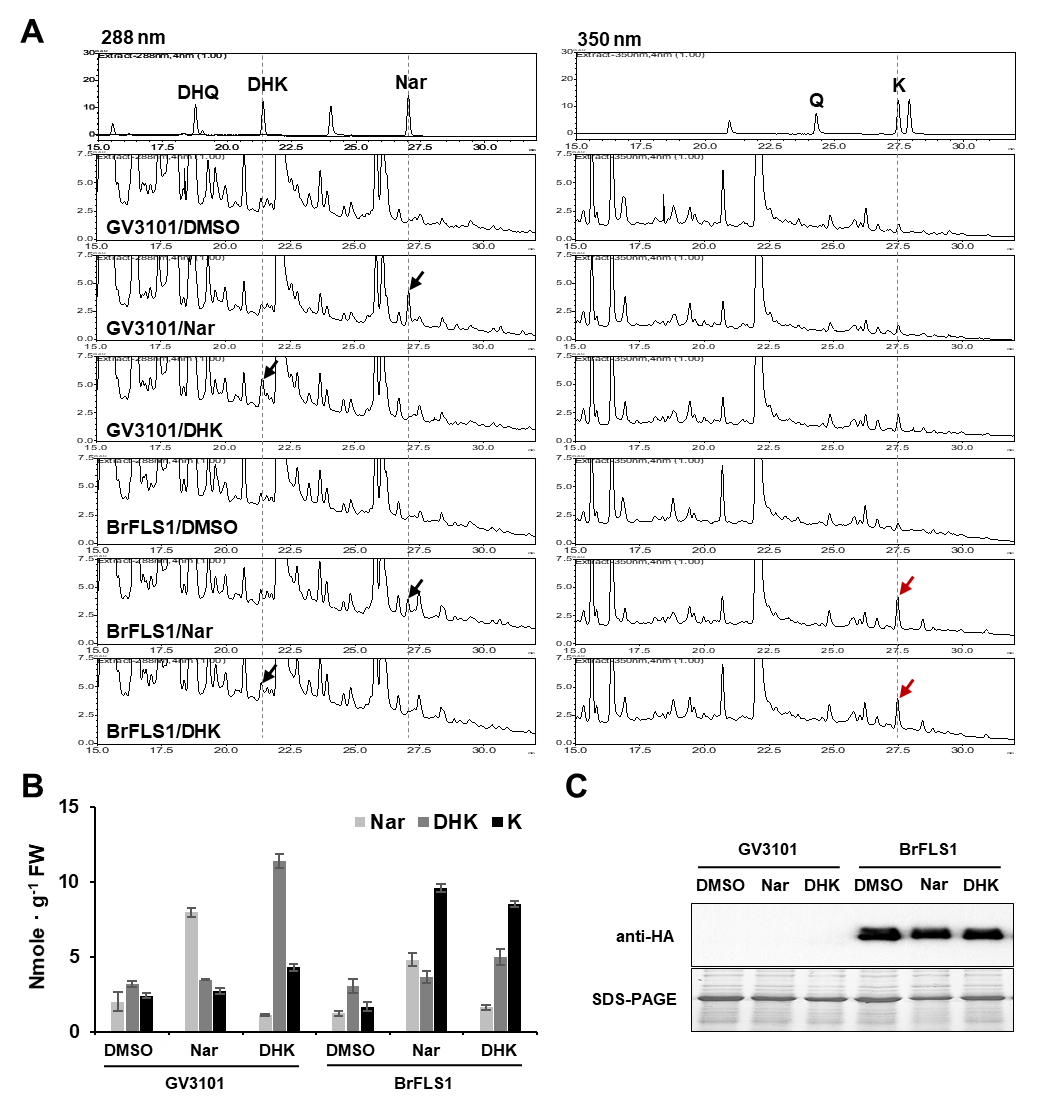
**

**Figure S3. Verification of BrFLS1 enzyme activity *in planta***

(A) Agrobacterium GV3101 with or without pB2GW7-*BrFLS1* construct was infiltrated into the *N. benthamiana* leaves. After four-day incubation, Nar or DHK was infiltrated into the same leaves as a substrate, and DMSO infiltration was conducted in parallel as a negative control, followed by 24 h incubation. Acid-hydrolyzed extracts from the leaves were subjected to HPLC analysis. Nar and DHK were detected at 288 nm and K was detected at 350 nm. Peaks were identified by comparing retention times and UV spectra of respective standards. Peaks corresponding to the infiltrated Nar and DHK are indicated by black arrows, and those corresponding to K products are indicated by red arrows. (B) Nar, DHK, and K contents in each sample were calculated based on the areas of corresponding standards. The mean values were determined from three replicates. (C) Western blot with anti-HA antibody verified the accumulation of BrFLS1 in the infiltrated leaves. SDS-PAGE was carried out for loading control.


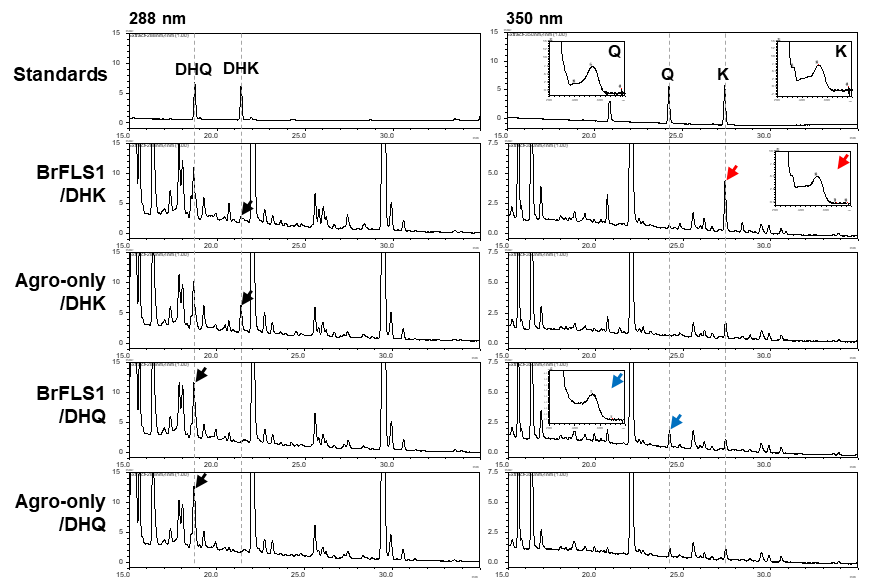


**Figure S4. Transient assay of BrFLS1 with different dihydroflavonol substrates**

Agrobacterium GV3101 with or without pB2GW7-*BrFLS1* construct were infiltrated into the *N. benthamiana* leaves. After four days, DHK or DHQ was infiltrated into the same leaves and incubated for 24 h. Leaf samples were extracted by acid hydrolysis and subjected to HPLC analysis. Dihydroflavonols (DHQ and DHK) were detected at 288 nm and flavonols (K and Q) were detected at 350 nm. Peaks were identified by comparing retention times and UV spectra of respective standards. Specific UV spectra of flavonols are displayed in the insets. Peaks corresponding to the infiltrated dihydroflavonol substrates are indicated by black arrows, and those corresponding to K and Q products are indicated by red and blue arrows, respectively.

**
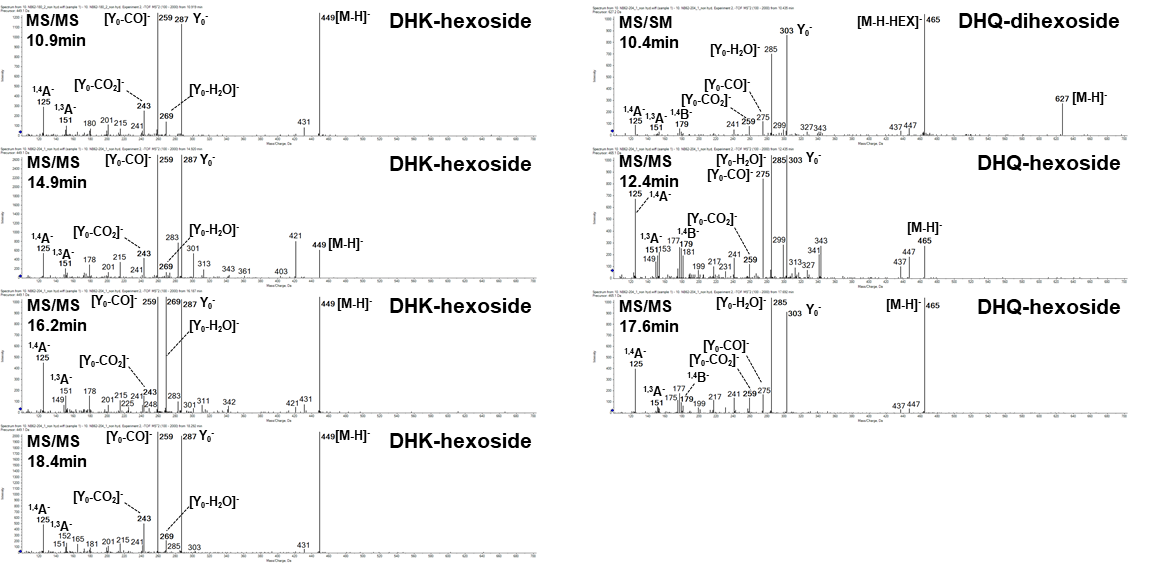
**

**Figure S5. Identification of dihydroflavonol-glycosides in the *brfls1* T_2_ plants by LC-ESI-QTOF-MS analysis**

LC-ESI-QTOF-MS/MS spectra of dihydroflavonol-glycosides corresponding to four major peaks in the XIC at *m/z* 287.25 (10.9, 14.9, 16.2, and 18.4 min) and three major peaks in the XIC at *m/z* 303.25 (10.4, 12.4, and 17.6 min) arising from the *brfls1* plants. The former are assigned as DHK-hexosides and the latter as DHQ-dihexoside or DHQ-hexosides.

**
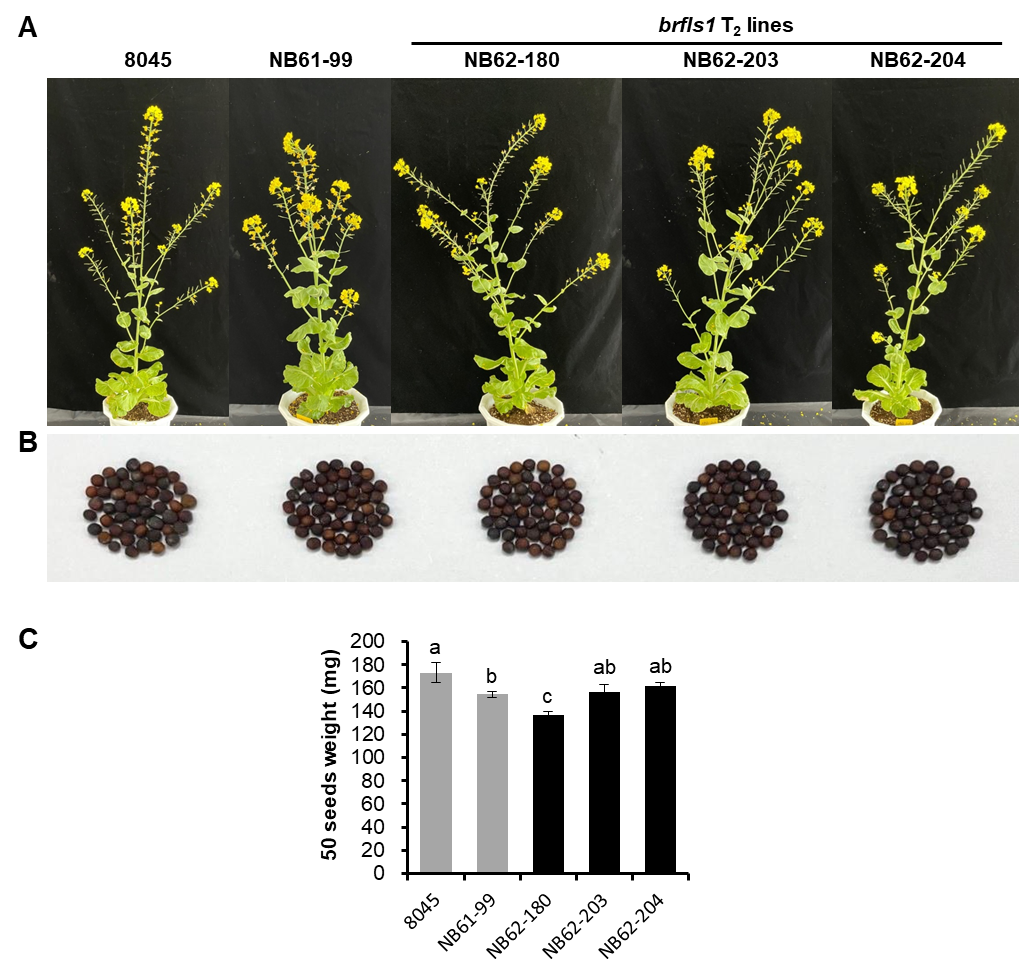
**

**Figure S6. Reproductive phenotypes of variety 8045, NB61-99, and *brfls1* T_2_ lines.**

Comparisons of phenotypes of (A) bolting and flowering, (B) seed-coat color and (C) seed weight between *brfls1* T_2_ lines and controls (8045 and NB61-99). Error bars indicate ±SD from three replicates. Statistical significance was determined by Duncan's Multiple Range Test using SAS (version 9.1) software. Significant differences between means (*p* < 0.05) are indicated by different lower-case letters (a,b, and c).

**
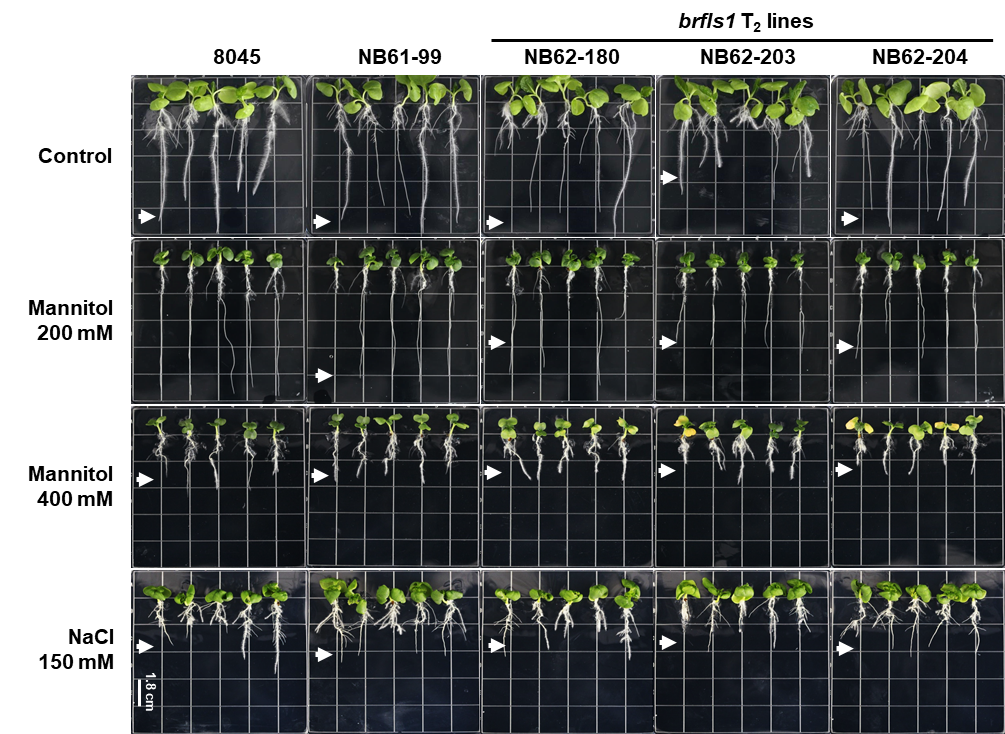
**

**Figure S7. Analysis of osmotic stress tolerance of the *brfls1* T_2_ seedlings.**

Seedlings of veriety 8045, NB61-99, and the *brfls1* T_2_ lines grown vertically on MS agar media with or without osmolytes (200 mM or 400 mM of mannitol or 150 mM of NaCl). Seedlings 4-day after germination were transferred to the media and cultured for four (for Control) or six days (for mannitol or NaCl treatment), and then their root lengths were measured. The white arrow represents the average root lengths on each plate.

**
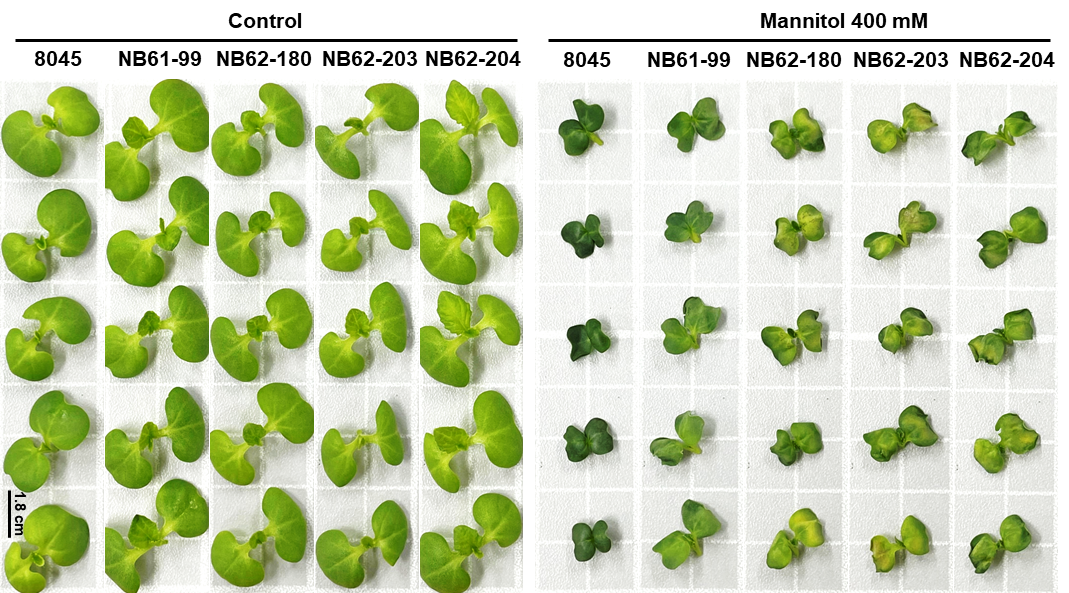
**

**Figure S8. Changes in chlorophyll contents of the *brfls1* T_2_ seedlings upon osmotic stress.**

Aerial parts of seedlings of veriety 8045, NB61-99, and the *brfls1* T_2_ lines grown on MS agar media with or without osmolytes (400 mM of mannitol).

**
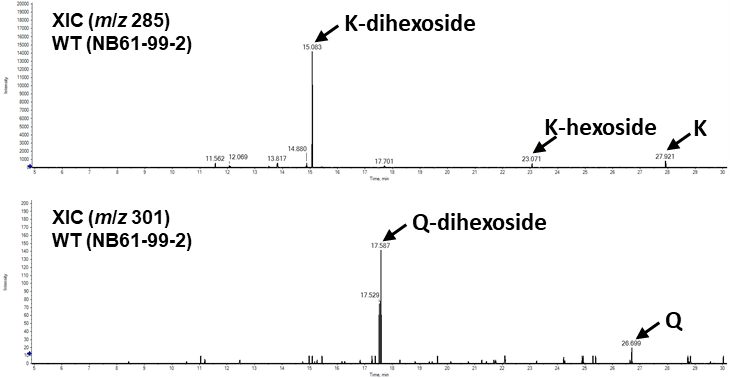
**

**Figure S9. LC-ESI-QTOF-MS analysis of flavonol-glycosides and aglycones in NB61-99-2**

Flavonol glycosides and residual aglycones in the NB61-99-2 (WT) were analyzed by LC-ESI-QTOF-MS with negative ionization mode. The XIC at m/z 285 corresponding to deprotonated K aglycone shows three peaks identified as K-dihexoside, K-hexoside, and K aglycone at 15.08, 23.07, and 27.95 min, respectively. The XIC at m/z 301 corresponding to deprotonated Q aglycone showes two peaks identified as Q-dihexoside, and Q aglycone at 17.59 and 26.70 min, respectively.

**Table S1. Primers used for cloning, off-target analysis, and qPCR**

| Gene | Locus ID | Purpose | Forward (5′ to 3′) | Reverse (5′ to 3′) |
| --- | --- | --- | --- | --- |
| *BrFLS1* | Bra009358 | CDS Cloning | ATGGAGATCGAGAGAGTCCAAGAC | ATTCATCAACACTTTTCTTCATGGTTTAGA |
|  |  | pGEX-cloning | GGGGCCCCTGGGATCCATGGAGATCGAGAGAGTCG | GGAATTCCGGGGATCCTCAGTCCAGAGGAAGCTTA |
|  |  | pENTR-cloning | CACCATGGAGATCGAGAGAGTCCAAG | CAACCCAAGCTTTCTTACCTTCTACTTC |
|  |  | Off-target analysis | GGTGGTGAAGGCGAGTGAAG | TTCTGTAGCTTCGTTCCGTACC |
| *BrFLS2* | Bra038647 | CDS Cloning | ATGGAAGTCAAGAAAGACCAGCAC | TCAATCGACCGGAAGCTTGAAAAGC |
|  |  | pGEX-cloning | GGGGCCCCTGGGATCCATGGAAGTCAAGAAAGACC | GGAATTCCGGGGATCCTCAATCGACCGGAAGCTTG |
|  |  | Off-target analysis | AGCACACATCTCCACCGTC | GCCCAAACCTCTCCTTCTGTATAC |
| *BrFLS3.1* | Bra038648 | CDS Cloning | ATGGACGTCCCTATCGTCGAC | TCAGTCGAGAGGAAGCTTCTTAAGC |
|  |  | pGEX-cloning | GGGGCCCCTGGGATCCATGGACGTCCCTATCGTCG | GGAATTCCGGGGATCCTCAGTCGAGAGGAAGCTTC |
|  |  | Off-target analysis | ATGGACGTCCCTATCGTCGAC | CTAGATCTTTCTGGTACTTGGTTCTG |
| *BrFLS3.2* | Bra029211 | Off-target analysis | GAGTTAGTCCCCTCGAAGTTCC | TGATCACAAAATTCTTATACTCGTGCC |
| *BrFLS3.3* | Bra029212 | Off-target analysis | GAATCAGTCCCTTCAAAGTTCCAAG | GGAACAATTACAACATTTTAATACTTGTACC |
| *BrFLS4.1* | Bra037747 | Off-target analysis | TTGAGAGAGACCAAACTGTATCCTC | TTGTCTTCTACATCTTGTTGGCTG |
| *BrFLS4.2* | Bra018076 | CDS Cloning | ATCCACCAGTACTAGCTACCAGC | ACTTTAATCATGAGTCGAGATCAACG |
|  |  | pGEX-cloning | GGGGCCCCTGGGATCCATGGTGGCTGAGAGAGACC | GGAATTCCGGGGATCCTCATGAGTCGAGATCAACG |
|  |  | Off-target analysis | TACCCATAATTGACCTAAGCAATCCC | CTAGGTCTAATGCGTACCTCCTCG |
| Off-target | Bra007696 | Off-target analysis | TCGAGATCTTCTACAACATGGCG | AAGCGTCGAGTAAATCAAGGGC |
|  | Bra027106 | Off-target analysis | AATACGCGGAGGCCATGAG | TCCTTCATGCATTGATCAAAGCTC |
|  | Bra039927 | Off-target analysis | ACGATGAACTTGGTCGAGACATC | TCTTCAAGAACGCTTGCTTTATCAG |
|  | Bra013222 | Off-target analysis | GAAGCTCAACAACAAGATCTCAGC | CGATTGCTGCTATGGCCATTG |
| *SpCas9* |  | Genotyping | GAGTTCTACAAGTTCATCAAGCC | TCGTTGTACACGGTGAAGTACTC |
| *Hyg-R* |  | Genotyping | AGCCTGAACTCACCGCGACGTCTGTCGAGAAGTTTC | GAGTTGGTCAAGACCAATGCG |
| *BrPAL1.2* | Bra017210 | qPCR | TGAGCAAGTCTACACGTACGC | CGAAGTCATTGCATTCTTCTCGC |
| *BrPAL2.1* | Bra006985 | qPCR | AGTGTTAACCACTGGAGTCAACG | GAGACAAGGATCATCCACGTACG |
| *BrPAL2.3* | Bra003126 | qPCR | CTCATCTCGTAAGACCTCAGAAGC | CTGTCTCAGATTCTCCTCAAGATGC |
| *BrPAL3.2* | Bra030322 | qPCR | GTCTGATCTCCAGCCACAAGAC | AGATGTCTTAGATCATAGGCTTGGC |
| *BrC4H1* | Bra018311 | qPCR | GCTTGCTGGCTACGACATC | TCATTACCGTTCGCTTCCACG |
| *BrC4H2* | Bra021636 | qPCR | AGACCATCCGTAGAAGAATGGC | CCAGGCATTGATCAAGATTCTGC |
| *Br4CL1* | Bra030429 | qPCR | GCTTCACACCGGAGATATCGG | CGTCGGTGATATCCTGATGGC |
| *BrCHS1* | Bra008792 | qPCR | GGCTCTCTTCAGTGACGGC | GCACCGTCAGAGTCTGGTAAG |
| *BrCHS3* | Bra023441 | qPCR | ACATGTCTAGCGCCTGTGTC | CTGTGTAGGACAACTGTCTCC |
| *BrF3H2* | Bra029996 | qPCR | ACCGTTACGTGAGGTGCTAG | TCATGGAAGCTTGATTGATTCGATAG |
| *BrF3′H* | Bra009312 | qPCR | AAGCACACGGACATGCTTAGC | CCGTACTTGCTGACGTGTCAG |
| *BrACT7* | XM_009127097 | qPCR | GTGGATATCAGGAAGGATCTGTATGG | CAGACACTGTACTTCCTCTCAGG |

**Table S2. Transcript per million (TPM) values of *BrFLS* homologs in Chinese cabbages**

| **Gene ID** | **Gene name** | **Chr.** | **5546-D9-1** | **5546-D9-2** | **5546-D9-3** | **5923-D9-1** | **5923-D9-2** | **5923-D9-3** | **5546-D42-1** | **5546-D42-2** | **5546-D42-3** | **5923-D42-1** | **5923-D42-2** | **5923-D42-3** |
| --- | --- | --- | --- | --- | --- | --- | --- | --- | --- | --- | --- | --- | --- | --- |
| **Bra009358** | **BrFLS1** | A10 | 34.36 | 31.79 | 33.39 | 16.64 | 17.31 | 17.39 | 6.11 | 7.57 | 7.35 | 14.61 | 16.04 | 18.15 |
| **Bra038647** | **BrFLS2** | A06 | 24.34 | 19.96 | 17.36 | 24.27 | 23.07 | 21.89 | 22.72 | 17.10 | 16.99 | 16.51 | 15.61 | 12.63 |
| **Bra038648** | **BrFLS3.1** | A06 | 3.67 | 3.96 | 1.92 | 2.69 | 3.81 | 4.86 | 8.33 | 8.97 | 5.91 | 5.31 | 4.75 | 6.89 |
| **Bra029211** | **BrFLS3.2** | A02 | 0.25 | 0.46 | 0.80 | 0.08 | 0.00 | 0.00 | 0.00 | 0.00 | 0.18 | 0.11 | 0.34 | 0.00 |
| **Bra029212** | **BrFLS3.3** | A02 | 2.24 | 2.13 | 2.14 | 0.18 | 0.59 | 0.41 | 0.89 | 0.12 | 1.41 | 0.46 | 0.75 | 0.49 |
| **Bra037747** | **BrFLS4.1** | A09 | 0.92 | 1.25 | 1.59 | 0.00 | 0.00 | 0.00 | 0.18 | 0.25 | 0.41 | 0.00 | 0.00 | 0.00 |
| **Bra018076** | **BrFLS4.2** | A06 | 7.91 | 6.15 | 7.49 | 4.49 | 3.01 | 3.39 | 2.10 | 4.64 | 5.29 | 2.62 | 3.10 | 2.13 |

**Table S3. Summary of acquisition process of *brfls1* plants**

| T_0_ Line | sgRNA | Indel freq.  in T_0_ (%) | Number of T_1_ seeds | Transgene-free  /tested | Heterozygous T_1_  /Transgene-free | Homozygous T_1_  /Transgene-free | Number of T_2_ seeds |
| --- | --- | --- | --- | --- | --- | --- | --- |
| NB60 | sg3 | 81.6  *(A ins.,45.2%;  T ins., 24.5%) | - |  |  |  |  |
| NB61 | sg3 | 11.3  *(A ins.,2.9%;  T ins., 3.3%) | 110 | 27/46 (58.7%) | 4/27 | 0/27 |  |
| NB62 | sg3 | 99.8  *(A ins.,44.9%;  T ins., 43.6%) | 400 | 6/171 (3.5%) | 0/6 | 5/6  (1/6: biallelic) | NB62-180: 556 |
|  |  |  |  |  |  |  | NB62-203: 234 |
|  |  |  |  |  |  |  | NB62-204: 140 |
| NB63 | sg3 | 0.2 |  |  |  |  |  |

^*^indicates frequencies of single bp insertions mainly occurred.

**Table S4. Off-target analysis for potential off-targets and *BrFLS* homologs**

| Category | Gene ID | Sequence | Number of mismatch | Region | Off-target  mutation |
| --- | --- | --- | --- | --- | --- |
| Potential off-target | Bra007696 | CATGGTGTTCGAGCTTCCGTGGG | 4 | Exon | Not detected |
|  | Bra027106 | AACGGTCTTCAAGCTTTCGTGGA | 3 | Exon | Not detected |
|  | Bra039927 | AACGTTCATTGAGCTTTTGTCGA | 4 | Exon | Not detected |
|  | Bra013222 | AAAGTCCTTCGAGCTTCCTCCCG | 4 | Exon | Not detected |
| *BrFLS* homologs | BrFLS2 | ACAGTTCTTCGAGCTACCGGAGA | 4 | Exon | Not detected |
|  | BrFLS3.1 | AAAGTTCTTCGAGCTTCCCGAGA | 3 | Exon | Not detected |
|  | BrFLS3.2 | AGAGTTCTTTGAGCTGCCTGAGA | 6 | Exon | Not detected |
|  | BrFLS3.3 | TGAGTTCTTTGAGCTGCCTGAGA | 7 | Exon | Not detected |
|  | BrFLS4.1 | ACATTTCTTTGAGCTCTCATCAG | 7 | Exon | Not detected |
|  | BrFLS4.2 | GCGATTCTTCGAGCTTTCTGAAG | 7 | Exon | Not detected |

Sequences mismatched with the sg3 target site are shown in red, and sequences corresponding to adjacent PAM are underlined.
